# Residual learning-based robotic image analysis model for low-voltage distributed photovoltaic fault identification and positioning

**DOI:** 10.3389/fnbot.2024.1396979

**Published:** 2024-04-22

**Authors:** Xudong Zhang, Yunlong Ge, Yifeng Wang, Jun Wang, Wenhao Wang, Lijun Lu

**Affiliations:** ^1^State Grid Hebei Electric Power Company, Shijiazhuang, China; ^2^Henan XJ Metering Co., Ltd., Xuchang, China

**Keywords:** low-voltage distributed photovoltaics, photovoltaic identification, positioning technology, unmanned aerial vehicle imagery, horizontal comparison experiment

## Abstract

With the fast development of large-scale Photovoltaic (PV) plants, the automatic PV fault identification and positioning have become an important task for the PV intelligent systems, aiming to guarantee the safety, reliability, and productivity of large-scale PV plants. In this paper, we propose a residual learning-based robotic (UAV) image analysis model for low-voltage distributed PV fault identification and positioning. In our target scenario, the unmanned aerial vehicles (UAVs) are deployed to acquire moving images of low-voltage distributed PV power plants. To get desired robustness and accuracy of PV image detection, we integrate residual learning with attention mechanism into the UAV image analysis model based on you only look once v4 (YOLOv4) network. Then, we design the sophisticated multi-scale spatial pyramid fusion and use it to optimize the YOLOv4 network for the nuanced task of fault localization within PV arrays, where the Complete-IOU loss is incorporated in the predictive modeling phase, significantly enhancing the accuracy and efficiency of fault detection. A series of experimental comparisons in terms of the accuracy of fault positioning are conducted, and the experimental results verify the feasibility and effectiveness of the proposed model in dealing with the safety and reliability maintenance of low-voltage distributed PV systems.

## 1 Introduction

In recent years, the Photovoltaic (PV) energy has experienced a fast development process, and increasingly, it plays an important role in our daily life due to the advantages of easy installation, cleaning, reasonable return period, and short construction period (Akram et al., 2020; Maka et al., 2021). Compared to conventional power generation approaches, PV power generation shows more superiorities such as safety, reliability, noiselessness, environmentally friendliness, resource distribution, and has a high energy quality and short construction period (Ali et al., 2020; Stiubiener et al., 2020). Especially, with the increasing energy demand, large-scale PV installations have received a surge of attentions, and this leads to the establishment of a mature PV market and technological innovation in the PV industry (Ali et al., 2020; Ma et al., 2022b). With the rapid development of large-scale PV power plants, automatic identification and localization of PV system faults are of critical importance to improving the safety, reliability, and productivity of PV systems. Faults in PV systems can reduce system efficiency and pose potential safety hazards, necessitating the proactive detection of potential faults and the implementation of appropriate corrective measures.

Typically, a large-scale PV system contain massive solar modules, which are variously interconnected with each other. Once one of them fail to work well, the efficiency of the PV system will be largely degraded. Therefore, it is urgently needed to actively detect any potential faults such that suitable corrective measures can be employed before the disruptions happen. To reach this goal, various artificial intelligence (AI) and robotic techniques have been utilized in the fault positioning/diagnosis of PVPP to enhance the intelligent processing capability (Ma et al., 2022b; Wang et al., 2024). Especially, due to the advantages of mobility, flexibility, programmability and large-area coverage, the unmanned aerial vehicles (UAVs) have been widely employed in the PV plant to capture moving images of various PV modules in the distributed power plants. Through intelligent image analysis, we can inspect specific PV faults in an efficient way, which is key to improve the operation and maintenance (O&M) level of the power plant (Atsu et al., 2020; Chen et al., 2020; Abubakar et al., 2021; Navid et al., 2021; Cui et al., 2022).

In fact, many deep learning models have been utilized for the improvement of PV-system fault diagnosis, such as the residual neural network, convolutional neural network, and semi-supervised ladder network (Atsu et al., 2020; Aziz et al., 2020). However, how to develop an effective AI-based approach, which can fully exploit useful fault feature information in UAV images, still is a challenging issue to enhance the fault diagnosis performance.

Regarding the on-site deployment of UAVs for inspection purposes, data used for inspection should be collected and processed first, including geographic information, environmental features, and specific requirements of the expected task. According to the characteristics of the target scene and the requirements of the task, the flight route, inspection frequency and data collection method of the UAV should be designed and implemented. The quality of PV images is challenging to guarantee, and issues such as under-exposure, high noise, and blurred details are frequently encountered. Moreover, the diversity of PV faults and the significant difference in image features among different faults pose significant challenges to the performance of image analysis algorithms.

In this paper, we propose a residual learning-based UAV image analysis model for low-voltage distributed PV fault identification and positioning. In our target PV power plants, the UAVs are deployed to acquire moving images of low-voltage distributed PV products. Based on YOLOv4 network, we integrate residual learning with attention mechanism into the UAV image analysis model, aiming to improve the robustness and accuracy of PV image detection. Then, we propose a sophisticated multi-scale spatial pyramid fusion method and use it to optimize the YOLOv4 network for the nuanced task of fault localization within PV arrays, where the Complete-IOU loss is used in the predictive modeling phase, which is able to significantly enhance the accuracy and efficiency of fault detection. To augment the size of the dataset, this paper adopts data augmentation techniques to flip and adjust the original samples. The image data used comes from three real power plants. The experimental results on a series of datasets verify the effectiveness of the proposed model.

The contributions of this paper are as follows:

1. Novel residual learning-based UAV image analysis model: A residual learning-based UAV image analysis model is proposed for PV fault recognition, where the residual learning network is constructed based on attention mechanism. This model is able to well exploit useful fault feature from UAV moving images, and then significantly boosts the accuracy and efficiency of PV fault identification and localization.
2. Enhanced YOLOv4 optimization for precise fault localization: An improved optimization method is designed to optimize YOLOv4 network, where a sophisticated multi-scale spatial pyramid fusion aims to optimize the model for the nuanced task of fault localization within PV arrays, while the Complete-IOU loss is used in the predictive modeling phase to enhance the accuracy of fault diagnosis in PV systems.
3. Extensive validation on real-world datasets: The proposed model has been trained and tested on a set of datasets to detect its application capability of PV fault detection and diagnosis.

The rest of the paper is organized as follows: section 2 reviews the related work on PV identification and fault positioning research. Section 3 describes the proposed methods in detail. Section 4 reports the experimental result and analysis. Section 5 represents the conclusion and future work.

## 2 Related work

Currently, many deep learning techniques have been successfully applied to real-world scenarios and effectively solved various challenging problems. For example, Jin et al. (2024) proposed a physical-informed neural network model with model predictive control controller and a perturbation-resistant neural dynamics controller equipped with the noise-suppression ability (Liufu et al., 2024). These two models addressed the challenges faced by autonomous vehicle control systems when dealing with unpredictable external environments and internal system noise disturbances. In the field of PV identification and fault localization, deep learning also has many applications.

Photovoltaic identification and fault localization are important components of PV O&M. It aims to timely discover and eliminate various faults in PV systems, thus improving the performance and safety of systems. Possible faults in PV systems include open-circuit faults, short-circuit faults, hot spot faults, etc. PV faults can be classified according to their occurrence time, severity, persistence, and cause, such as infant failures, midlife failures, wear-out failures, acute failures, chronic failures, permanent failures, and temporary failures (Hong and Pula, 2022). These faults will cause the output power of PV systems to decrease, and even lead to serious consequences such as fire. Therefore, effective methods are needed to diagnose and locate faults in PV systems.

Fault detection methods based on electrical data are a type of method that uses historical or real-time data of voltage, current, power, etc. in PV systems to extract feature information through data mining, machine learning and artificial intelligence technologies. This feature information is then used to establish fault identification and location classifiers or regressors. Some researchers used data analysis techniques such as feature extraction, clustering, classification, and regression to estimate the faults location and severity of PV arrays based on the voltage and current data in PV systems (Alajmi and Abdel-Qader, 2016; Chen and Wang, 2017; Fadhel et al., 2019; Patil and Hinge, 2019). Fadhel et al. proposed a framework for PV faults detection and localization based on hybrid data, which combines voltage–current sensor network and environmental sensors, such as temperature, humidity, and irradiance. It improves the accuracy and robustness of PV faults detection and localization (Alajmi et al., 2018). These methods have the advantages of being independent of model parameters and adaptable to complex environmental conditions. However, it also has the disadvantages of requiring additional sensors, instruments and circuits, and being dependent on environmental conditions.

Computer vision-based fault detection methods use artificial intelligence technologies such as deep learning to process and interpret large amounts of visible or infrared images in PV systems, thus achieving the automation and intelligence of PV fault identification and localization. Some researchers proposed a PV fault detection and localization method based on convolutional neural networks. This method used the thermal imaging data of PV systems to achieve the detection and localization of open circuit faults, short circuit faults, hot spot faults, and shadow faults in PV arrays through image processing, feature extraction, and classifier training (Ali et al., 2020; Herraiz et al., 2020; Alves et al., 2021). Korkmaz et al. proposed a PV fault classification method based on transfer learning and multi-scale convolutional neural networks. This method used the visible light image data of PV systems to achieve the classification of open circuit faults, short circuit faults, hot spot faults, and shadow faults of PV modules through image preprocessing, feature extraction, and classifier training (Korkmaz and Acikgoz, 2022). These methods have the advantages of being able to handle complex nonlinear problems, as well as having self-adaptation and learning ability. However, they also have the disadvantages of requiring a lot of training data, computing resources and algorithm optimization.

This section provides an overview of research concerning photovoltaic identification and fault localization. It primarily explores fault types, diagnostic methods, localization techniques and deep learning models related to photovoltaic modules. However, existing fault Identification and positioning techniques for PV systems based on current location suffer from poor robustness, especially when environmental conditions change. Computer vision-based fault Identification and positioning methods may not achieve the expected accuracy when the data are insufficient or the training is inadequate. In addition, the processing range of image processing methods is narrow, which may not cover all types of faults in PV systems. In response to these limitations, we propose methods of photovoltaic identification and fault localization in low-voltage distributed PVPP.

## 3 Methods

**Figure 1:**
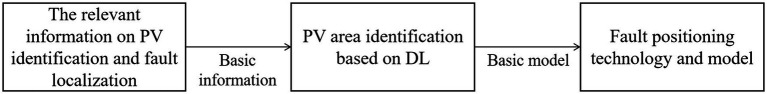
Methodological framework of the study.

This section elaborates on the methods of photovoltaic identification and fault localization in low-voltage distributed PVPP, emphasizing the integration and optimization of deep learning technology, especially in the context of drone imaging. The structure of the method presented in this paper is shown in Figure 1.

The primary aim of this framework is to enhance photovoltaic identification and fault localization using deep learning techniques, in order to improve the operational efficiency and safety of photovoltaic power plants. First, the relevant information of low-voltage distributed photovoltaic power plants is acquired. Then, the deep learning model undergoes analysis, and the identification model earmarked for optimization is determined. The model is improved by combining residual U-shaped module composed of dilated convolution and residual network, achieving accurate identification of the photovoltaic area. Finally, the photovoltaic fault localization technique is refined using multi-scale spatial pyramid fusion, Complete-IOU loss and self-attention mechanism, thereby achieving precise fault positioning in photovoltaic power plants.

### 3.1 PV identification and fault positioning of low-voltage distributed PVPP

**Figure 2:**
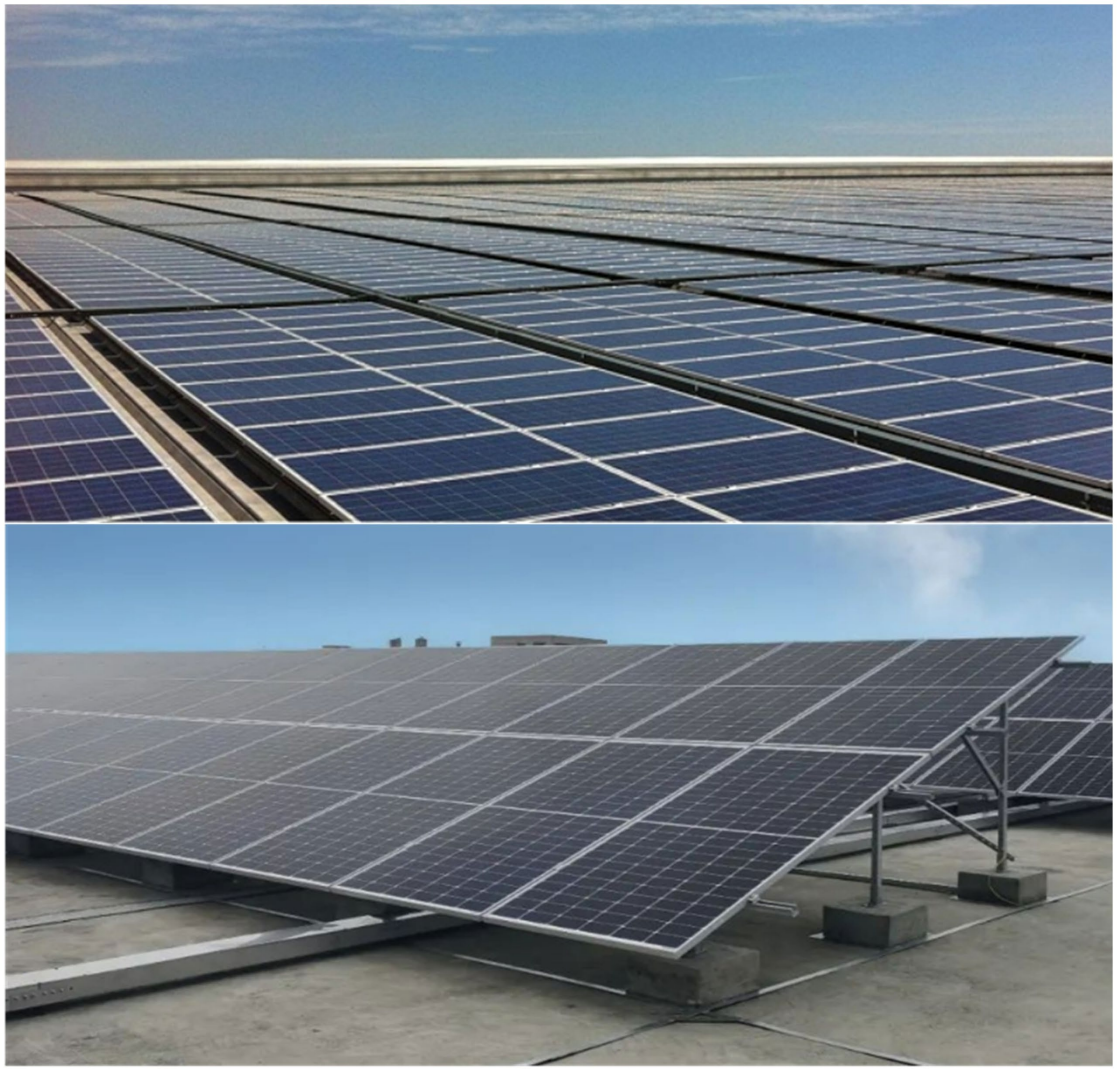
Real view of low-voltage distributed PVPP.

A PVPP is a facility that uses light energy to convert it into electricity. It is composed of solar panels and inverters. Solar panels convert sunlight into DC electricity. Inverters convert DC energy into alternating current energy, which is fed into the grid. PVPPs can be divided into centralized and distributed according to their nature. Distributed PVPPs are often installed in factories, residential roofs, fish ponds, and other small ground or building areas, generally connected to the grid through 380 V voltage. It operates flexibly and independently of the grid under the right conditions (Gallardo-Saavedra et al., 2020). The advantages of PVPPs include no emissions, low maintenance costs, and long life. It can effectively reduce environmental pollution and energy consumption. However, the disadvantage is that it depends on the weather and light intensity, and the construction and maintenance costs are relatively high (Gao et al., 2021). As a PV system with rapid growth in installed capacity in the next few years, more and more PV power distribution and installation urgently need efficient and low-cost PVPP health inspection methods to detect the function of PV modules and ensure the normal operation of the system (Jie et al., 2020). Figure 2 shows the actual picture of the low-voltage distributed PVPP.

In a complete PV-EG system, after connecting several PV modules in series, a PV string with DC output is formed, which is called a string. The PV brackets used in PVPP can be divided into fixed and tracking brackets. The tracking brackets can automatically adjust the direction to maximize production capacity (Khalil et al., 2020). In the traditional PV bracket unit design, the PV modules are arranged in vertical double rows or horizontal three and four rows. A bracket unit is usually equipped with one or two strings. The number of modules is determined by the number of modules connected in series in the PV string. PV modules as the core EG module, from top to bottom: glass, upper encapsulant, cell, lower encapsulant, backplane, frame, and junction box (Li et al., 2021).

**Figure 3:**
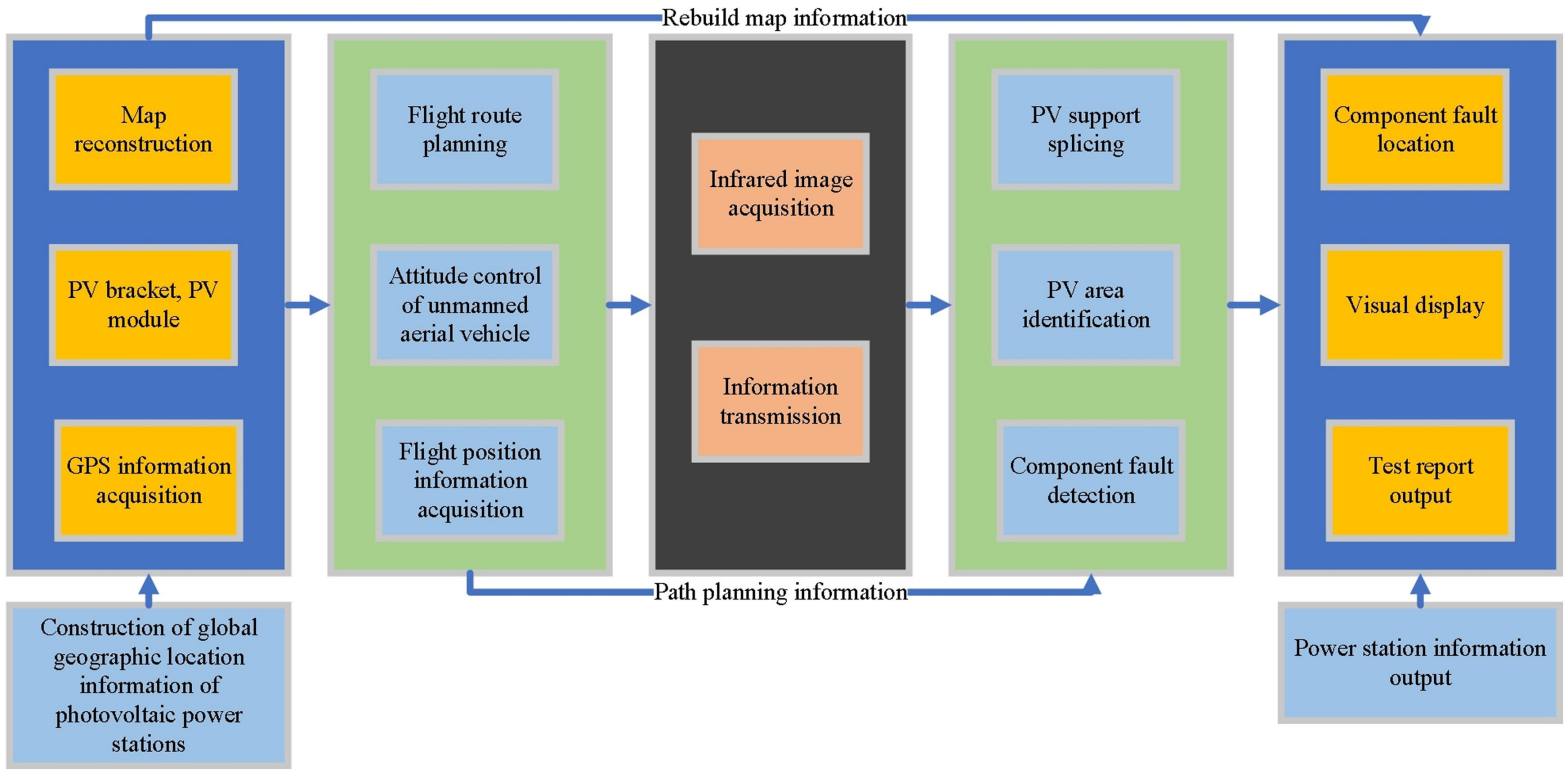
UAV infrared image fault diagnosis process.

When a hot spot fails on the cell, highlighted areas appear in the infrared image. Therefore, for defect detection, infrared image defect diagnosis is one of the most widely used defect detection methods. Infrared thermal imaging cameras can detect faults caused by internal defects in PV modules and judge the severity of the fault based on the temperature in a non-invasive way. Inspection UAVs can obtain information, including altitude and temperature, by integrating UAVs, sensors and infrared cameras (Liang et al., 2020a). The detection process is shown in Figure 3.

### 3.2 PV area identification based on DL

Photovoltaic identification identifies important information, such as the brand and model of the solar panel, by extracting and classifying the features of the solar panel. PV identification plays an important role in the assessment, supervision, and management of PVPPs. Traditional PV identification identifies the transmitted back image through UAV. For most infrared images obtained by PVPP, the PV area absorbs more heat and has a more obvious temperature difference from the ground. The grayscale histogram will have obvious peaks. The PV area can be obtained using a suitable gray threshold method (Liang et al., 2020b). For single-peaked prominent pictures, simple pre-and post-scene separation is done. The maximum inter-class variance threshold segmentation algorithm is considered to find the best threshold for segmentation (Liu et al., 2020). The principle is to convolve the original image through a convolution check of a specific shape and size. When a point pixel of the original image and its surrounding pixels can form a convolution kernel shape, it is retained. If not, it is deleted. The expansion operation, on the contrary, can be used to enlarge the objects in the image or recombine the separated pixels (Lyu et al., 2020).

**Figure 4:**
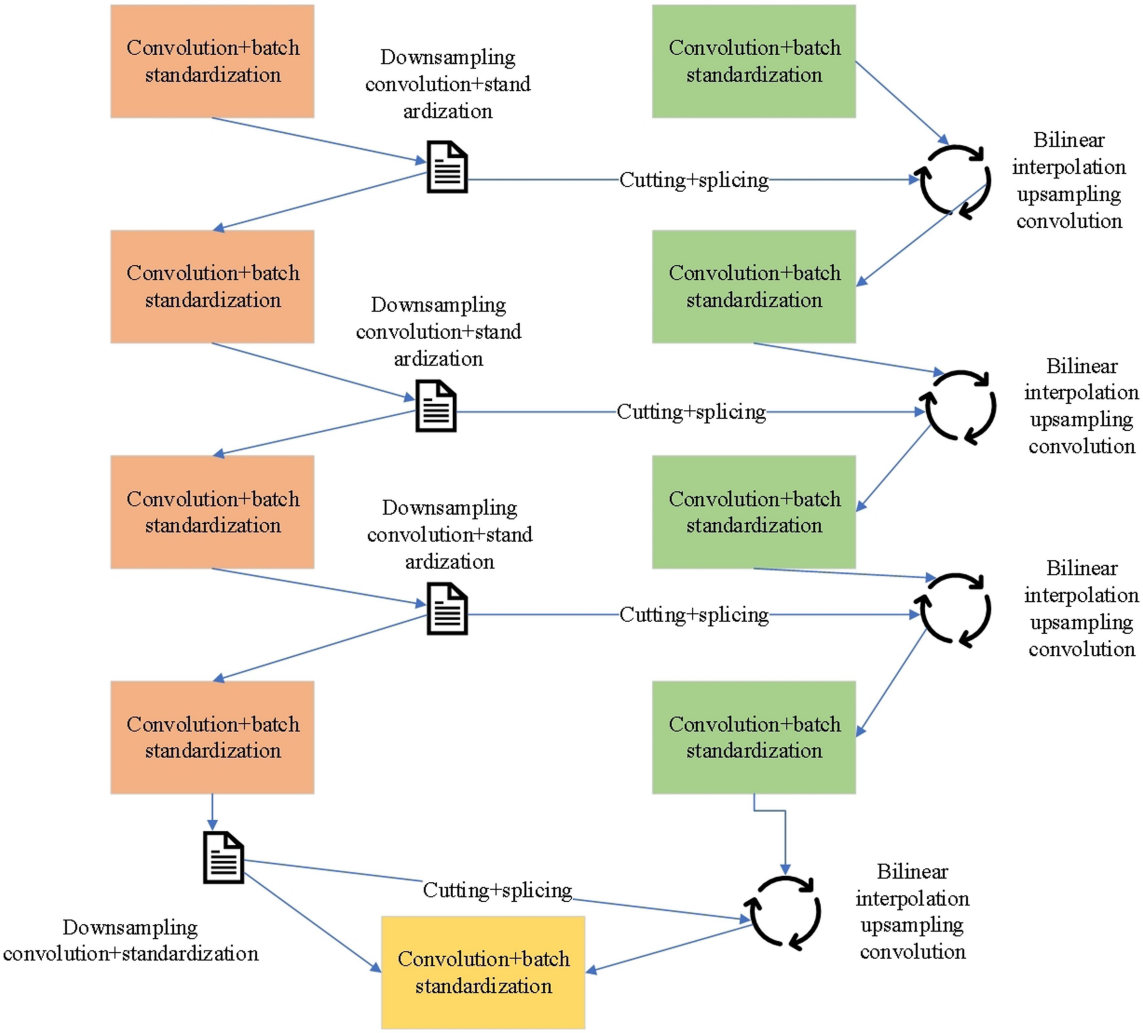
Net semantic segmentation model.

For traditional image processing algorithms, the process is intuitive, and the calculation is simple and effective. However, the processing scenario is single, and the robustness is low. When the ambient temperature and the PV area temperature are similar, or there is interference in the environment, it is difficult to determine the threshold by the maximum inter-class variance threshold segmentation algorithm in the grayscale histogram. The algorithm directly fails (Manno et al., 2021). The PV region in the infrared image has a typical visual salience, which can be regarded as a binary pixel classification problem in semantic segmentation. The purpose of the semantic segmentation task is to correctly classify each pixel of an image. Each pixel can be classified into a corresponding category by manually determining the semantic label of each pixel and learning (Marqusee et al., 2021). In the PV area visual recognition task, the pixels in the infrared image can be divided into two categories. The target pixel is the PV area pixel, and all the remaining pixels are classified as the background. So, the U-Network (U-Net) semantic segmentation model is introduced. Figure 4 displays the structure of the U-Net model.

The left side of Figure 4 is the downsampling part. It is found that the feature map decreases, and the number of channels increases. The same convolution kernel on the right side is upsampled using the bilinear interpolation method. At present, the U-Net model is affected by the size of the convolution kernel, resulting in small receptive fields and incomplete information capture. Therefore, the receptive field is increased by pooling and dilated convolution. The pooling operation is accompanied by decreased resolution while increasing the receptive field. Dilated convolution can increase the receptive field without increasing the number of parameters and reducing the convolution kernel.


(1) $$k^{\prime }=k+\left(k-1\right)*\left(d-1\right)$$

(2) $$RF_{i+1}=RF_{i}+\left[\left(k^{\prime }-1\right)*\overset{i}{\underset{i=1}{\prod }}\mathrm{Strid}e_{i}\right]$$

In Equations (1, 2), k is the convolution kernel size, and $k^{\prime }$ is the dilated convolution kernel size. d is the dilation rate hyper-parameter. $RF_{i}$ is the receptive field of the previous layer. $RF_{i+1}$ is the current receptive field. $\overset{i}{\underset{i=1}{\prod }}\mathrm{Strid}e_{i}$ is the stride product, and *i* is the number of layers. As the network deepens, degradation and gradient vanishing problems may occur, resulting in deep neural networks that are difficult to train. Although many studies have optimized network architectures, the problem has not been fully solved yet (Li et al., 2023). So, residual learning is introduced. Residual learning is easier than direct learning, so errors can be calculated through residual learning.


(3) $$F\left(x\right)=H\left(x\right)-x$$

**Figure 5:**
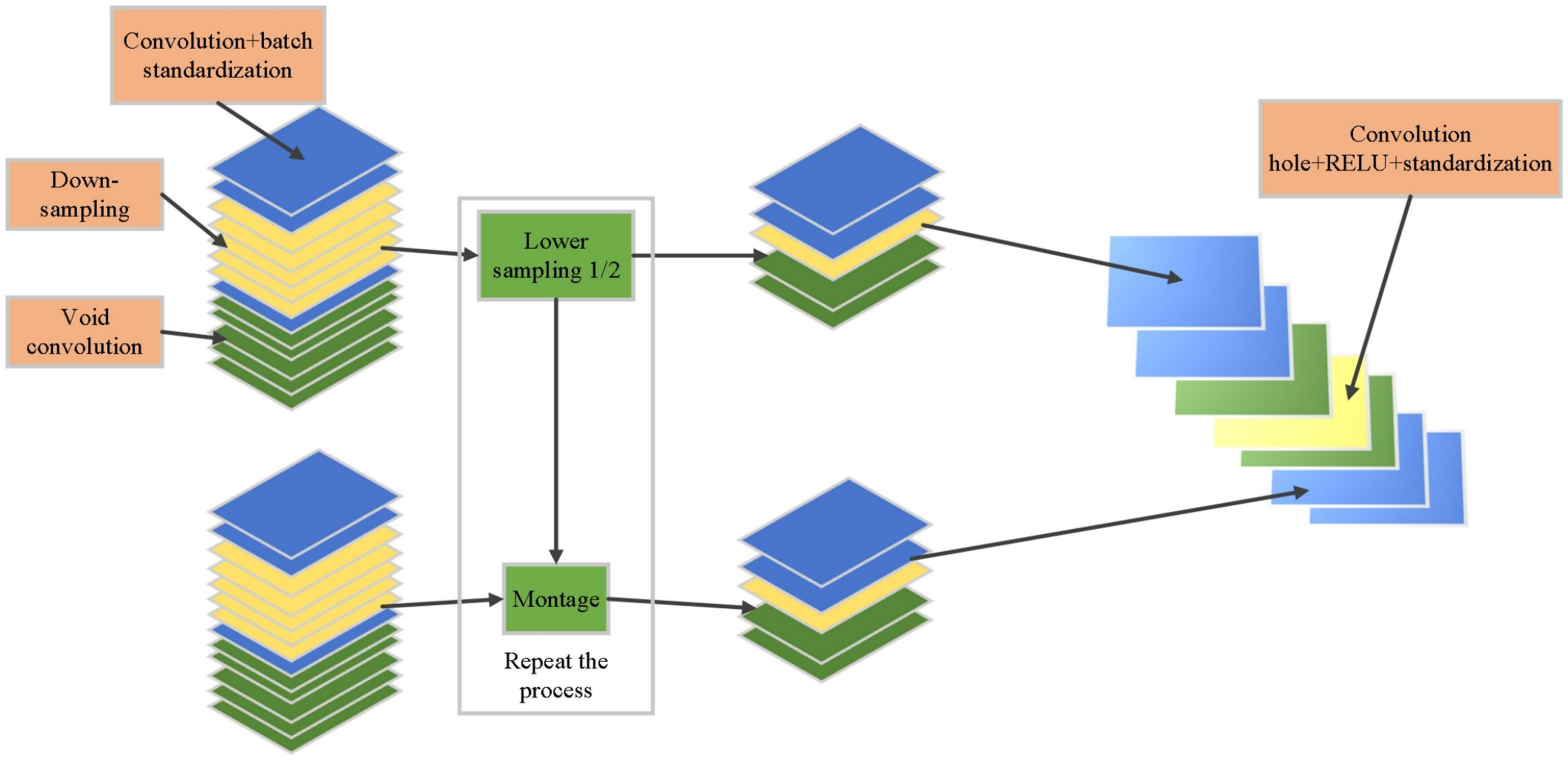
Optimized semantic segmentation model.

In Equation (3), x is the input information, $F\left(x\right)$ is the result of residual learning, and $H\left(x\right)$ is the result of direct learning. Deeper structures under reasonable calculation can be constructed through the residual U-shaped module composed of dilated convolution and residual network to obtain multi-scale features. The residual U-shaped module retrieves edge features through feature joining, alleviating the problem that the receptive field of simple convolution is too small to capture global information. The overall structure of the optimized model is shown in Figure 5.

Its main architecture is a classical U-Net network similar to Encoder-Decoder, a simple and effective saliency object detection network that can be used for the semantic segmentation of two classifications. Each stage comprises residual U-shaped modules, which can more effectively capture the global features of multi-scale features. Specific residual U-shaped modules are used in different encoder and decoder stages. From the first layer to the seventh layer, the information structure of the picture has been analyzed and deconstructed. Residual learning enables the model to construct deeper structures and extract multi-scale features with reasonable computation, which facilitates effective visual identification of PV areas under various power plant scenarios and environmental interferences. It also empowers the model with better adaptability and learning ability when dealing with complex non-linear problems. The advantage of this is that more and more feature information will be extracted. The key components of the optimized model is shown in Table 1.

**Table 1:** The key components of the optimized model.

Layer number	Component	Description
1	Input layer	Initial layer receiving the input image.
2	Encoder stage 1	First stage of the encoder with residual U-shaped module for initial feature extraction.
3	Encoder stage 2	Second stage of the encoder with enhanced residual U-shaped module for deeper feature analysis.
4	Intermediate layer	Central layer of the network, bridging encoder and decoder, with complex feature integration.
5	Decoder stage 1	First stage of the decoder, reconstructing features and integrating global information.
6	Decoder stage 2	Second stage of the decoder, refining the feature reconstruction and detail enhancement.
7	Output layer	Final layer producing the segmented image output, representing the analyzed and processed features.

The process of the proposed optimized semantic segmentation model can be as follows:

Step 1: The input image is fed into the input layer of the network for feature extraction.
Step 2: In encoder stage 1, the residual U-shaped module performs a preliminary feature analysis on the image, extracting low-level edge and texture information.
Step 3: In encoder stage 2, the enhanced residual U-shaped module conducts a deeper feature analysis on the image, extracting high-level semantic and structural information.
Step 4: At the intermediate layer, a complex feature integration method combines features at different scales and resolutions, resulting in a global feature representation.
Step 5: In decoder stage 1, bilinear interpolation upsampling and convolution operations reconstruct the features, while concatenation operations connect the features from encoder stage 1 with the features from decoder stage 1, achieving global information transfer.
Step 6: In decoder stage 2, bilinear interpolation upsampling and convolution operations further reconstruct the features, while concatenation operations connect the features from the input layer with the features from decoder stage 2, achieving detail information recovery.
Step 7: At the output layer, convolution operations transform the features into a binary semantic segmentation image, representing the analyzed and processed features.

### 3.3 Fault positioning technology and model of low-voltage distributed PVPP

For the infrared image fault diagnosis system of PVPP based on UAV inspection, the health of the components is the most concerned item of the power plant after obtaining infrared images. The corresponding PV module information can be used to guide subsequent O&M after making judgments in infrared fault diagnosis (Mellit and Kalogirou, 2021). In the UAV inspection proposed here, the shooting height can be set in the path planning stage. The field of view of the infrared picture can be locked by collecting the original power plant information and camera angle. The main PV mounts are adjusted horizontally to ensure *m* complete brackets in one infrared image or one complete bracket in *n* continuous images (Qais et al., 2020). For the resulting profile composed of several points, the vertex information can be obtained by quadrilateral fitting by the Ramer-Douglas-Peucker algorithm (Quiles et al., 2020). This method, also known as the iterative endpoint fitting algorithm, is an algorithm that approximates the curve as a series of points and reduces the number of points. The contour points obtained are a subset of the original contour (Ramadan et al., 2020).

The Line Segment Detector method is a linear detection method with low time complexity. It forms a horizontal line field by first calculating the horizontal line angle within the eight neighborhoods of each pixel. The vertical angle of the gradient direction of this pixel is the horizontal line angle, and the horizontal line field is a matrix corresponding to the points in the image one by one (Ridha et al., 2020). After obtaining the horizontal line field, the area growth method is used to generate several connected domains according to the horizontal line angle. The threshold *t* is set to 22.5 degrees, and the horizontal line angle change of all pixels in each connected domain cannot exceed *t*. The connected domain obtained at this time is called the line support area. Each line support area is a candidate for segment detection. Each line support area corresponds to a matrix, represented by its smallest circumscribed rectangle (Ridha et al., 2021). The line support area’s spindle direction is the matrix’s major axis direction, and the rectangle covers the entire area. The smallest external rectangle represents the straight-line information. The confirmation line can be obtained by filtering the fitted rectangle information. The complete single PV module can be obtained by simple area extraction (Romero-Fiances et al., 2022).

**Figure 6:**
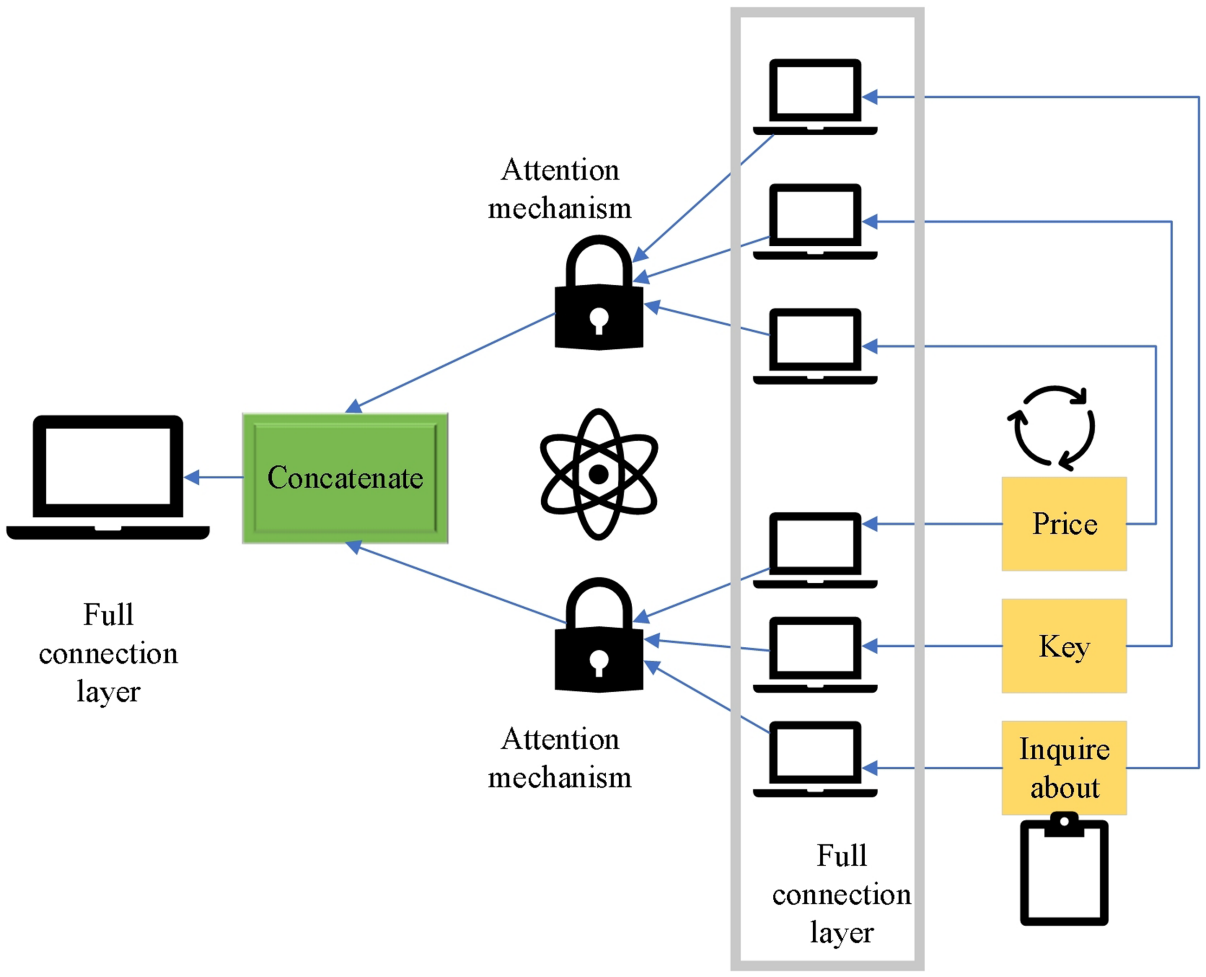
Multi-headed self-attention mechanism.

Common fault positioning classification models are convolutional neural networks (CNNs) and Transformer, and the dominant CNNs benefit from their inductive bias. For example, the adjacent area has adjacent and translation invariance characteristics. The implied visual prior knowledge can be effectively used to extract information. Therefore, there is good ingestion even for small data, although many studies have been conducted to optimize the CNN architecture (Ma et al., 2023a), which may still lead to performance degradation (Tsanakas et al., 2020). In sequence-related tasks in natural language processing (NLP) neighborhoods, recurrent neural networks (RNNs) with fixed structure memory units simplify the difficulty of long-distance learning and significantly outperform other RNNs (Veerasamy et al., 2021). However, with the introduction of the attention mechanism, it broke through the field of traditional NLP and became the model with the best performance. Figure 6 demonstrates the structure of multi-headed self-attention mechanism.

Compared with CNNs, calculating the association between two positions in the AM only requires calculating the association weight between the two pairs. In contrast, multi-layer convolution is required in CNNs to obtain the relationship between distant positions. However, the model based on the self-attention mechanism needs to calculate more parameters and lacks the inductive bias brought by convolution. CNN has more advantages when there is less data. The target detection network is the dual information determination task of target position and category information (Yang et al., 2021). Traditional detection modes use a combination of candidate boxes and feature extraction. For example, manually designed features are extracted in each window by sliding windows. Then, the features are obtained with a simple classifier (Zghaibeh et al., 2022; Ma et al., 2022a, 2023b). Here, the you only look once v4 (YOLOv4) network is needed. Its overall loss function consists of regression box loss, confidence loss, and classification loss. A threshold is set for confidence. For lower thresholds, no classification loss occurs. This method can solve the problem of sample imbalance well, which is suitable for the detection target required here. The formula of Complete-IOU loss for YOLOv4 is shown below:


(4) $$L_{\mathrm{CIoU}}=1-IoU+\frac{\rho^{2}\left(b,b^{gt}\right)}{c^{2}}+\alpha v$$

(5) $$v=\frac{4}{\pi^{2}}{\left(\arctan\frac{w^{gt}}{h^{gt}}-\arctan\frac{w}{h}\right)}^{2}$$

(6) $$\alpha =\frac{v}{\left(1-IoU\right)+v}$$

**Figure 7:**
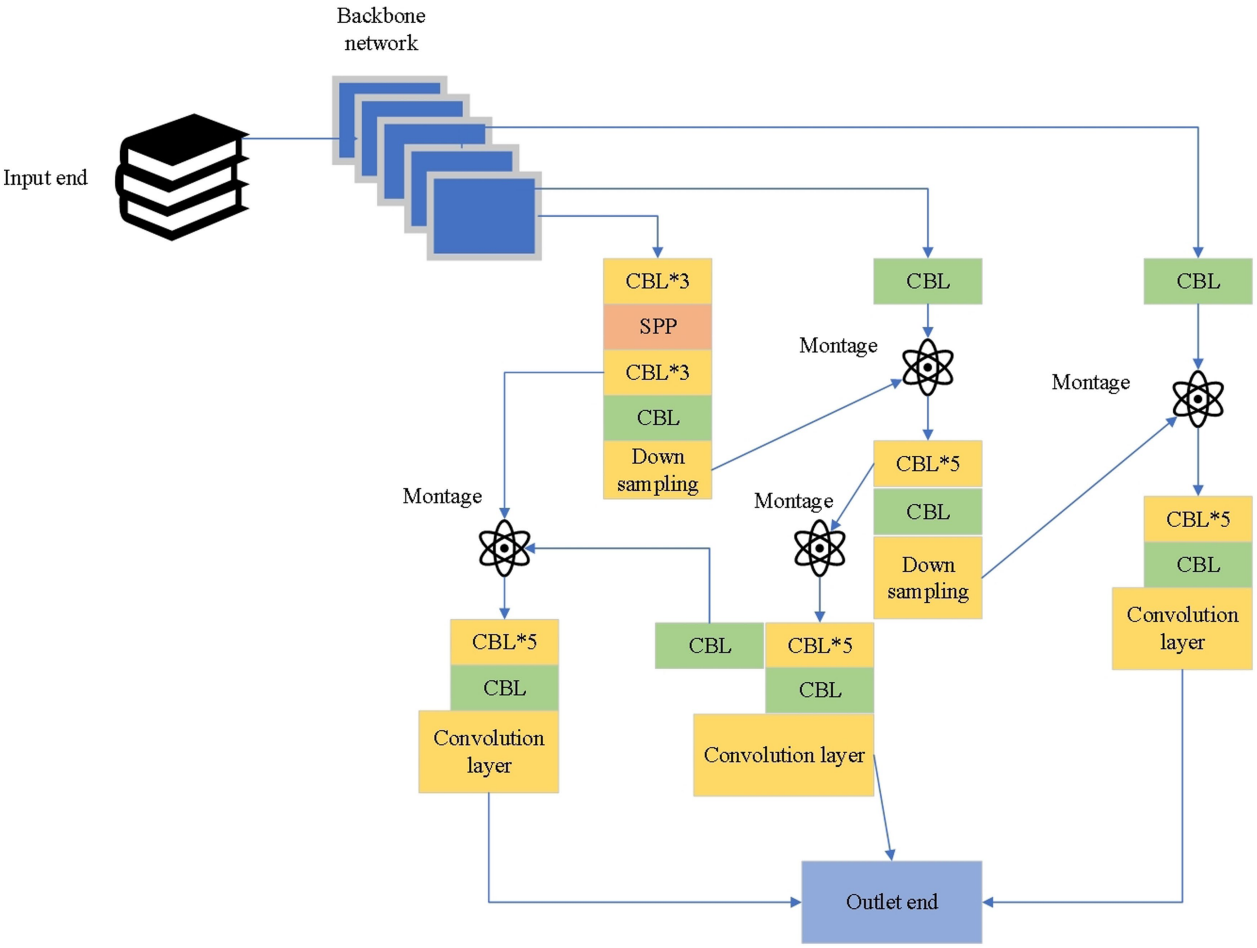
Optimized structure of YOLOv4.

In Equations (4–6), IoU is the overlap between the predicted bounding box and the ground truth bounding box. $\frac{\rho^{2}\left(b,b^{gt}\right)}{c^{2}}$ is the Euclidean distance between the centers of the predicted bounding box (b) and the ground truth bounding box ($b^{gt}$), normalized by the diagonal length of the smallest enclosing box covering both boxes. $c^{2}$ is the diagonal length of the smallest enclosing box covering both the predicted and ground truth bounding boxes. αv is a value that represents the aspect ratio difference between the predicted bounding box and the ground truth bounding box. The optimized YOLOv4 network structure is given in Figure 7.

Based on the optimized structure of YOLOv4 shown above, a PV positioning method of low-voltage distributed PVPP is proposed, and its process is as follows:

Step 1: The feature map obtained from the backbone network is fed into the prediction network. The CBL includes three components: convolution (Conv), batch normalization (Batch Norm), and leaky rectified linear unit (Leaky ReLU).
Step 2: Introduce additional layers in the prediction network to optimize the algorithm performance, enhance the feature detection capabilities, and increase the sensitivity to the nuances of the PV array images.
Step 3: After using a multi-scale fusion of spatial pyramid pooling (SPP), YOLOv4 improves the feature extraction capability through the feature pyramid and path aggregation network fusion structure. The feature pyramid network, in conjunction with the path aggregation network, forms a fusion structure that effectively consolidates features at various scales and resolutions.
Step 4: Complete-IOU loss is used in the prediction, which integrates the prediction box boundary non-coincidence, center distance information, and aspect ratio information so that the regression operation of the prediction box obtains fast speed and high accuracy. The utilization of Complete-IOU loss results in a more nuanced and detailed regression operation of the prediction box.

This enhances both the speed and accuracy of the algorithm, allowing for rapid processing without compromising the quality of fault detection. The accurate detection of boundaries, combined with the precise localization of faults, makes our optimized YOLOv4 algorithm particularly effective for PV fault positioning. This level of accuracy is critical in the context of PV maintenance, where the timely and precise identification of faults can significantly impact the efficiency and longevity of PV installations.

## 4 Experimental result and analysis

### 4.1 Comparative analysis of experimental results of PV area identification

The image data used in the experimental training set comes from three real power plants. They represent three types of power plants: mountain PV, fishery-solar complementary, and agro-solar complementary. They are named training set 1, training set 2, and training set 3. The test data set is also selected from three real power plants, called test set 1, test set 2, and test set 3. The original samples of the dataset are flipped and adjusted for broadening. We set the ratio of the training sets to the tests set at 8:2. The specific sample number of the dataset is shown in Table 2.

**Table 2:** PV identification dataset.

Power plant	Number of samples	Number of samples (data expansion)
Training set 1	120	1,200
Training set 2	195	1,950
Training set 3	494	4,940
Test set 1	97	X
Test set 2	42	X
Test set 3	43	X

The models participating in the comparative experiment include Otsu, Grabcut, U-Net, Recurrent Residual CNN-based U-Net (RES U-Net), U2-Net, and the optimized model proposed here. The structure and hyperparameter settings of the algorithm used in the experiment are shown in Table 3.

**Table 3:** Structure and hyperparameter settings.

Hyperparameter	Value
Initial learning rate	0.001
Momentum term	0.949
Convolution Kernel size (*k*)	5 × 5
Batch size	64
Dilation rate	3

**Figure 8:**
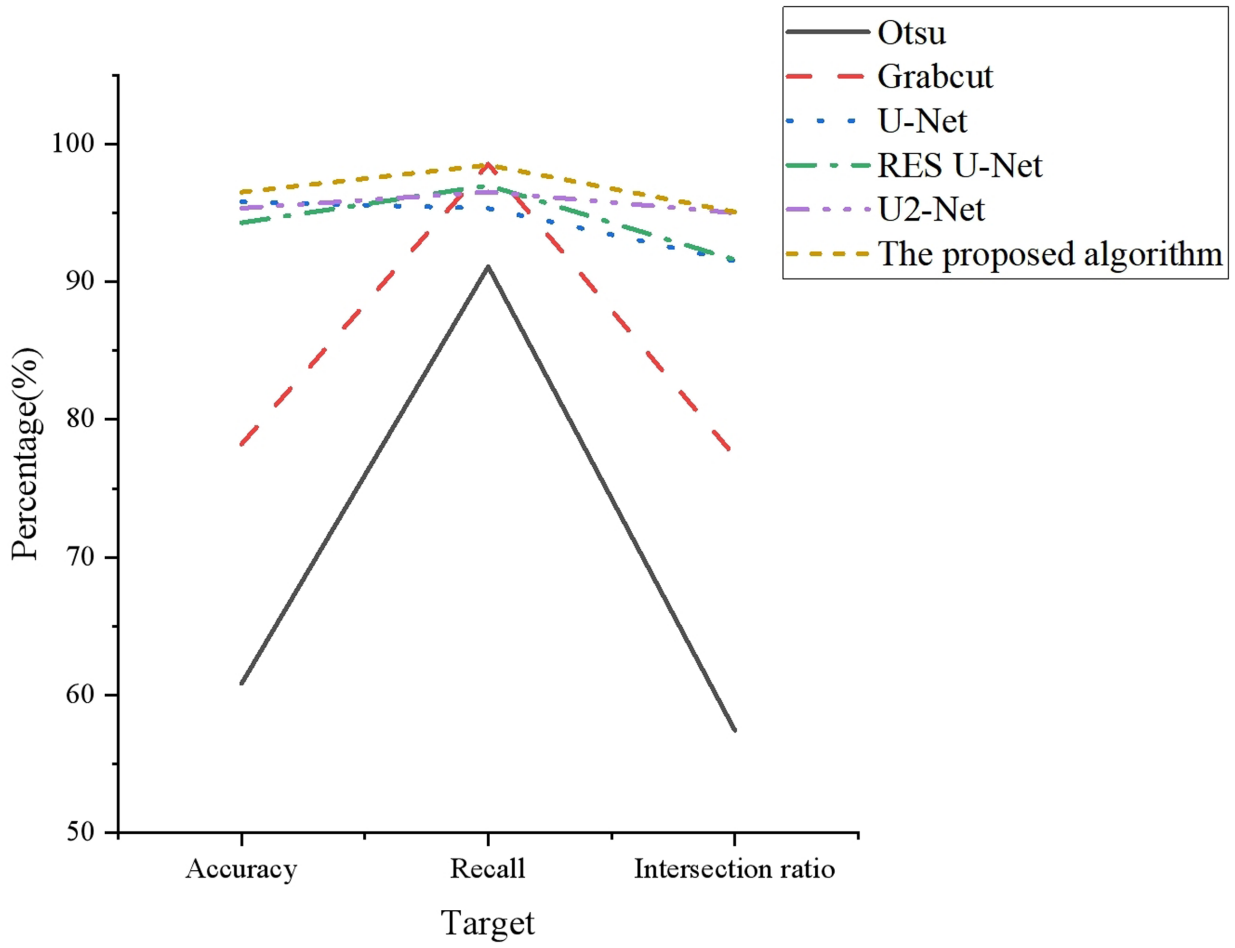
Statistics of identification results.

There are three experimental evaluation indicators: accuracy, recall, and Intersection over Union (IoU). Accuracy represents the proportion of correctly identified PV area pixels to the total pixels. Recall represents the proportion of correctly identified PV area pixels to the actual PV area pixels. IoU represents the ratio of the intersection of correctly identified PV area pixels and actual PV area pixels to their union. Figure 8 reveals the results.

From Figure 8, in the comparative experiments of accuracy and IoU, the corresponding data of the optimized model are the highest, 96.5 and 95.07%, respectively. This indicates that the model can effectively distinguish between the PV area and the background area, and has a high degree of matching with the real PV area. Regarding recall, the optimized model has the highest recall rate of 98.46%, except that it is not as high as the Grabcut model of 98.54%. Its recall rate is only 0.08% lower than the Grabcut model. This indicates that the model can cover most of the real PV areas, but there are also a few cases of missed detection. Comparative experiments can show that the optimized model has high adaptability to different power plant scenarios and environmental disturbances. Additionally, it has a high degree of attention to the data at the edge of the PV power plant, which can effectively carry out visual identification of the PV area from the image.

The optimized U-Network semantic segmentation model proposed here uses residual U-shaped module composed of dilated convolution and residual network, which can build deeper structures and obtain multi-scale features under reasonable computation. The model extract edge features through feature joining, avoiding the problems of gradient disappearance and degradation in neural network, and alleviating the problem of small receptive field and inability to capture global information in simple convolution. This can effectively achieve accurate identification of the photovoltaic area. The introduction of residual learning enhances feature extraction ability of the model, enabling the model to adapt to different power station scenarios and environmental disturbances, thus effectively performing visual recognition of the PV area.

Based on the above improvements, the proposed algorithm not only effectively addresses the limitations of limited receptive fields and inability to capture global information inherent in simple convolutions. More importantly, it exhibits remarkable adaptability in handling PV area identification tasks under diverse power plant scenarios and environmental interferences. This is the reason why the optimized model proposed in this aper can achieve good performance.

### 4.2 Experimental analysis of fault positioning in PV area

**Figure 9:**
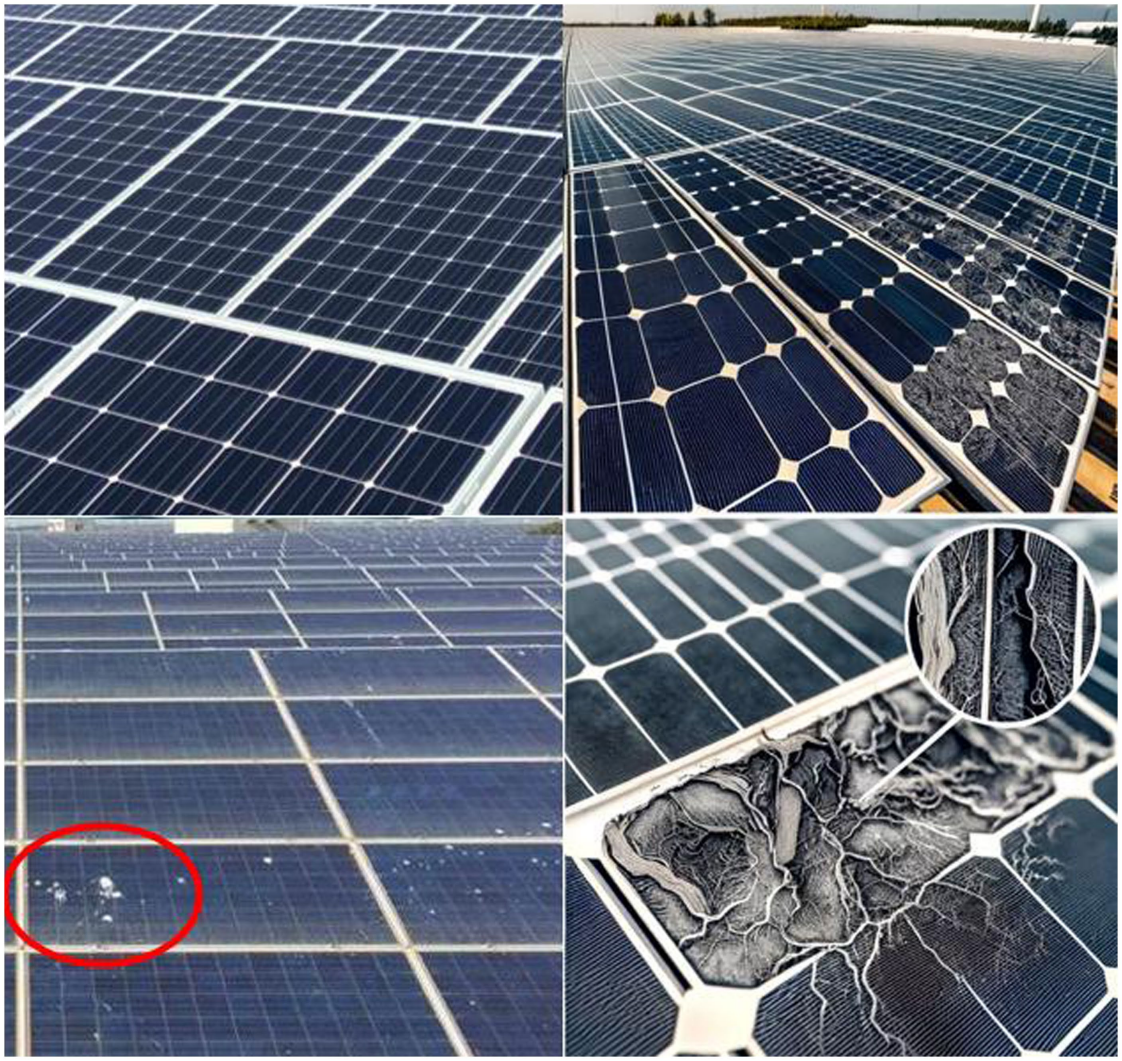
PV images in datasets.

The dataset of the fault positioning experiment uses the dataset in PV identification, from which 2,000 sheets are extracted to construct the training set. The PV images in datasets are shown in Figure 9.

The number of the fault and normal components is classified. The data of the target detection dataset is presented in Table 4.

**Table 4:** Target detection dataset.

Data set	Number of pictures	Number of normal components	Number of hot spot fault components	Number of diode conducting components
Training set	2,000	99,444	3,623	322
Test set	809	36,589	3,439	422

**Figure 10:**
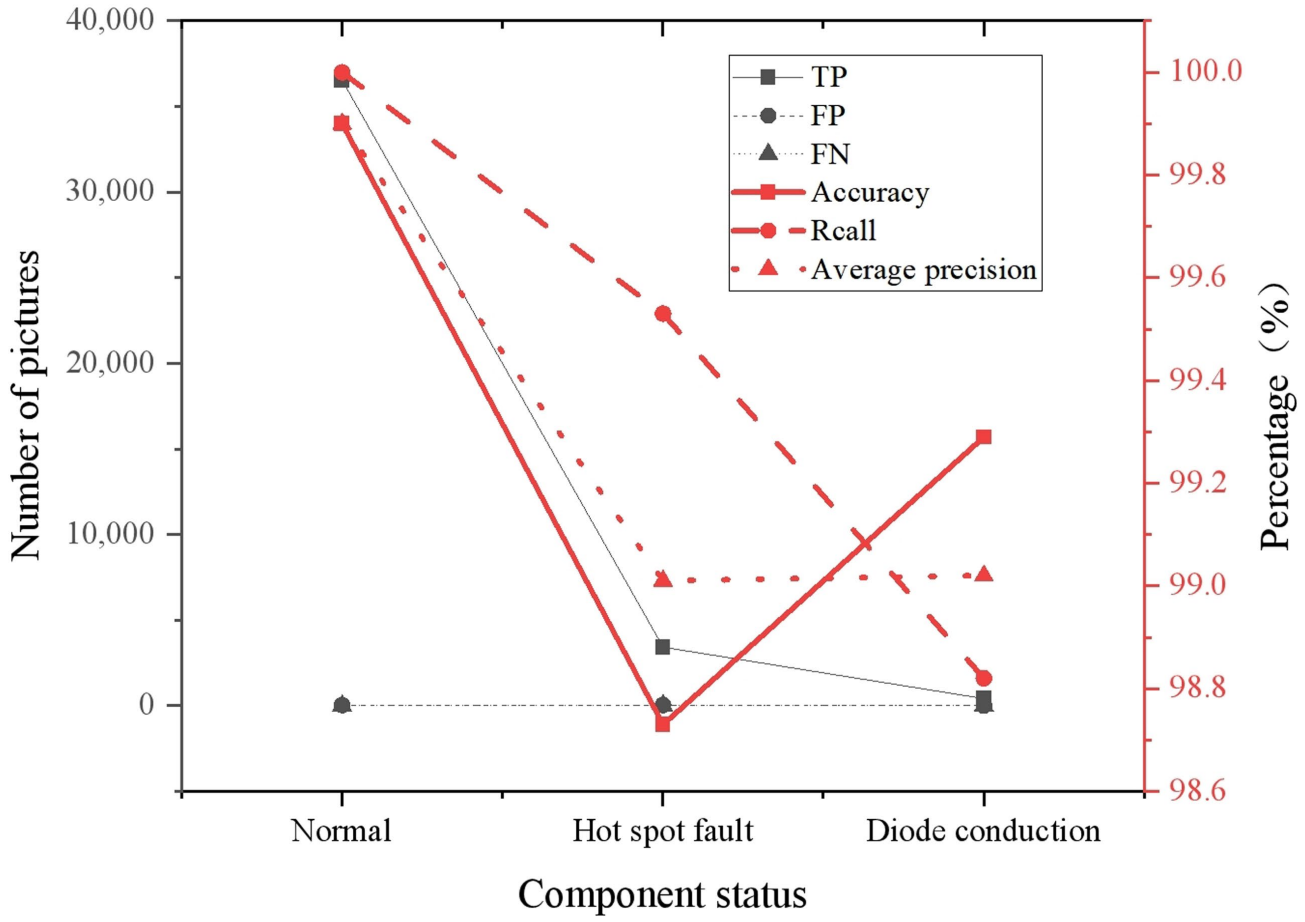
Statistics of positioning results.

The evaluation indicators are precision, recall, true positive (TP), false positive (FP), false negative (FN) and average precision. The experimental results are plotted in Figure 10.

**Figure 11:**
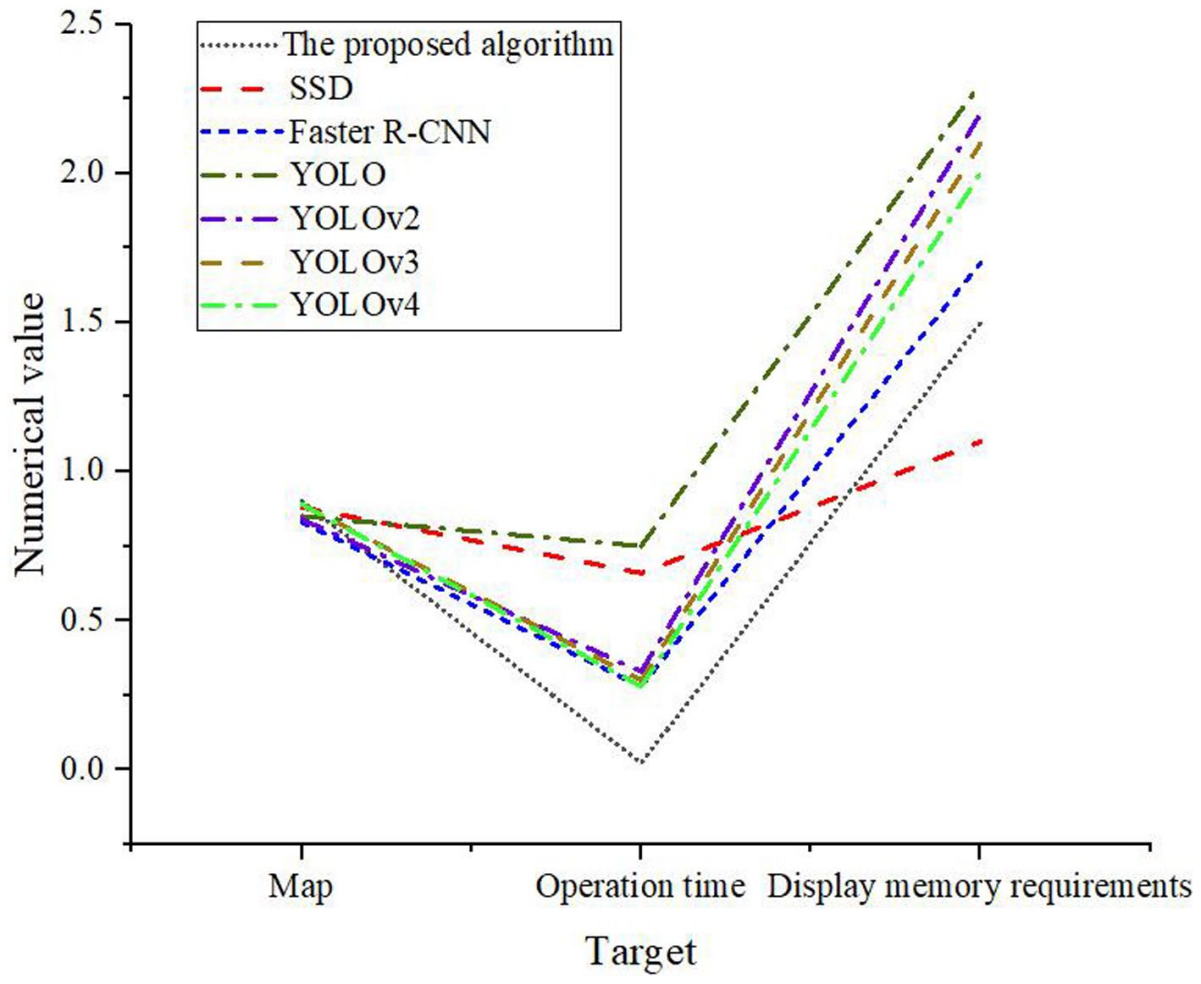
Horizontal comparison of the model.

From Figure 10, the optimized YOLOv4 has played a very high performance. The recall rate of normal components has reached 100%, and the precision has gained 99.9% by testing normal components. When testing hot spot fault components, it is found that the recall rate of hot spot fault components also reaches 99.53%, and the precision rate reaches 98.73%. In the identification of normal components, the data of FN is 0. In addition to high precision and recall, the proposed model has great advantages in terms of operation time. Models chosen for comparison include single shot multibox detector (SSD), Faster region based convolutional neural network (Faster R-CNN), YOLO, YOLOv2, YOLOv3, and YOLOv4. The experimental results are shown in Figure 11.

Through the comparison of Figure 11, the average accuracy (Map) of the optimized model is the highest, at 90%. The memory requirement is 1.5G, and its operation time is only 0.022 s. This shows that the optimized YOLOv4 model has lower computational complexity and higher running speed, which can quickly process a large number of UAV infrared images, saving O&M costs and time. Through horizontal comparison, the prediction time of the proposed model is shorter than that of other traditional models, and the memory requirement is small. This shows that the optimized YOLOv4 model occupies less resources and is more suitable for deployment and running on devices with limited resources. Combined with the previous experiments, the overall accuracy and recall of the optimized model are much higher than those of the traditional model for PV identification and fault positioning. Therefore, the rationality and effectiveness of the proposed model can be verified by comparative experiments.

From the above analysis, it is evident that the optimized YOLOv4 model has a significant advantage in the fault positioning task of PV components, not only performing well in terms of precision and recall, but also having obvious optimization in prediction time and memory requirement. The optimized YOLOv4 proposed here improves the robustness and generalization ability of the model by effectively integrating features of different scales and resolutions through multi-scale fusion of spatial pyramids. This can make the model to recognize both macro and micro features in PV array images. The Complete-IOU loss function takes into account the non-overlap, center distance and aspect ratio of prediction box. This can refine the regression process for prediction boxes, and enhance localization precision and recall rates, and facilitating precise fault localization in PV systems. These improvements enable the optimized YOLOv4 model to better capture and analyze the details and features of the PV component images, thereby enhancing the performance of fault localization.

Experimental results show that the proposed PV identification model has high accuracy and IoU. In PV identification, the accuracy rate of the optimized model can reach 96.5%, and the IoU is 95.07%. Among the six models compared, the IoU is also the highest. Moreover, the recall rate of the optimized model is only 0.08% lower than that of the Grabcut model, which has the highest recall rate. It verifies the effectiveness of the PV identification model proposed here. In the experiment of fault positioning, precision, recall, TP, FP, FN, and average precision are used as performance indicators. The fault positioning model achieves 100% recall and precision of 99.9% in testing normal components. For the hot spot fault component test, the recall rate of the hot spot fault component also reaches 99.53%, and the precision rate reaches 98.73%. The model performs well. Meanwhile, a horizontal comparison is added at the end of the experiment. Compared with the traditional model, the average accuracy value of the optimized model is the highest, 90%. The memory requirement is 1.5G, and its operation time is only 0.022 s, significantly exceeding the operation time of other models.

The experimental results demonstrate that the proposed model has high practical value and effectiveness in the O&M of real PV power plants. It can provide strong support for the safe, reliable and efficient operation of PV power plants. Specifically, the model can accurately locate faults in PV modules, helping O&M personnel to timely discover and solve problems, thereby improving the operation efficiency and long-term stability of PV power plants. For example, in the actual application of a certain PV power plant, the model successfully identified a batch of aging PV modules and replaced them in time, avoiding accidents at the power plant.

## 5 Conclusion

As the installed capacity of PV power generation increases rapidly, how to detect abnormalities and faults of PV modules in an efficient manner has become a key challenge to maintain the safety, reliability, and productivity of large-scale PV plants. With the consideration that all the fault information of the PV module exists in the moving images of UAVs, we propose an improved residual learning model to extract useful fault feature from the UAV moving images, and then use it for low-voltage distributed PV fault identification and positioning. This way works in an end-to-end way, and it can not only detect single faults, but also identify the existence of hybrid PV faults. First, we integrate residual learning with attention mechanism into the UAV image analysis model, aiming to improve the robustness and accuracy of PV image detection. Then, we propose a sophisticated multi-scale spatial pyramid fusion method for the optimization of the YOLOv4 network, targeting at the nuanced task of fault localization within PV arrays, where the Complete-IOU loss is used in the predictive modeling phase, significantly enhancing the accuracy and efficiency of fault detection. The proposed novel residual learning model and optimized YOLOv4 network were applied to fault identification and localization in low-voltage distributed PV systems. The models were trained and tested on a real-world dataset, demonstrating their application potential in fault detection and diagnosis of low-voltage distributed PV systems. This research is of great significance to ensure the safety, reliability and productivity of large-scale PV power plants.

The current training dataset is restricted to a limited number of fault categories, hindering direct applicability to fault identification and positioning of equipment in other types or domains. To enhance generalization capability and robustness of the proposed algorithm, we will focus on expanding the dataset to encompass a broader spectrum of fault types in future research. Moreover, considering that many novel and effective computer vision and deep learning methods emerge rapidly, we will adopt state-of-the-art intelligent models rather than the original neural network models in the target PV fault identification and positioning. In addition, we attempt to optimize the scheduling problem after fault localization using multi-objective optimization algorithms.

## Data availability statement

The raw data supporting the conclusions of this article will be made available by the authors, without undue reservation.

## Author contributions

XZ: Conceptualization, Formal analysis, Methodology, Validation, Writing – original draft, Writing – review & editing. YG: Formal analysis, Methodology, Validation, Writing – review & editing. YW: Data curation, Formal analysis, Validation, Writing – review & editing. JW: Formal analysis, Methodology, Validation, Writing – review & editing. WW: Data curation, Validation, Writing – review & editing. LL: Data curation, Validation, Writing – review & editing.
